# Treatment-seeking patterns for malaria in pharmacies in five sub-Saharan African countries

**DOI:** 10.1186/s12936-017-1997-3

**Published:** 2017-08-29

**Authors:** Joël Ladner, Ben Davis, Etienne Audureau, Joseph Saba

**Affiliations:** 1 Rouen University Hospital, Epidemiology and Health Promotion Department, Hôpital Charles Nicolle, 1 Rue de Germont, 76 031 Rouen Cedex, France; 2 Axios International, Paris, France; 3 Paris Est University Hôpital, Henri Mondor Hospital, Public Health, Assistance Publique Hôpitaux de Paris, Créteil, France

**Keywords:** Malaria, Treatment, ACT drugs, Private sector

## Abstract

**Background:**

Artemisinin-based combination therapy (ACT) is recommended as the first-line anti-malarial treatment strategy in sub-Saharan African countries. WHO policy recommends parasitological confirmation by microscopy or rapid diagnostic test (RDT) in all cases of suspected malaria prior to treatment. Gaps remain in understanding the factors that influence patient treatment-seeking behaviour and anti-malarial drug purchase decisions in the private sector. The objective of this study was to identify patient treatment-seeking behaviour in Ghana, Kenya, Nigeria, Tanzania, and Uganda.

**Methods:**

Face-to-face patient interviews were conducted at a total of 208 randomly selected retail outlets in five countries. At each outlet, exit interviews were conducted with five patients who indicated they had come seeking anti-malarial treatment. The questionnaire was anonymous and standardized in the five countries and collected data on different factors, including socio-demographic characteristics, history of illness, diagnostic practices (i.e. microscopy or RDT), prescription practices and treatment purchase. The price paid for the treatment was also collected from the outlet vendor.

**Results:**

A total of 994 patients were included from the five countries. Location of malaria diagnosis was significantly different in the five countries. A total of 484 blood diagnostic tests were performed, (72.3% with microscopy and 27.7% with RDT). ACTs were purchased by 72.5% of patients who had undergone blood testing and 86.5% of patients without a blood test, regardless of whether the test result was positive or negative (p < 10^−4^). A total of 531 patients (53.4%) had an anti-malarial drug prescription, of which 82.9% were prescriptions for an ACT. There were significant differences in prescriptions by country. A total of 923 patients (92.9%) purchased anti-malarial drugs in an outlet, including 79.1% of patients purchasing an ACT drug: 98.0% in Ghana, 90.5% in Kenya, 80.4% in Nigeria, 69.2% in Tanzania, and 57.7% in Uganda (p < 10^−4^). Having a drug prescription was not a significant predictive factor associated with an ACT drug purchase (except in Kenya). The number of ACT drugs purchased with a prescription was greater than the number purchased without a prescription in Kenya, Nigeria and Tanzania.

**Conclusions:**

This study highlights differences in drug prescription and purchase patterns in five sub-Saharan African countries. The private sector is playing an increasingly important role in fever case management in sub-Saharan Africa. Understanding the characteristics of private retail outlets and the role they play in providing anti-malaria drugs may support the design of effective malaria interventions.

## Background

Over the past decade there has been a renewed interest in research on malaria and innovations to tackle the disease, including diagnostic methods, drugs and the development of control measures [1]. Although reported cases of malaria decreased by 34% in Africa between 2000 and 2013 [2], the disease continues to be a major public health problem globally. Approximately 214 million cases of malaria occur annually and 3.2 billion people are at risk of infection [3]. In 2015, around 438,000 deaths were attributed to malaria, with an estimated 90% of these deaths reported in sub-Saharan African countries [4].

In 2006, the World Health Organization (WHO) recommended the first-line use of artemisinin-based combination therapy (ACT) to address the resistance of *Plasmodium falciparum* to monotherapy, and to improve malaria treatment outcomes [5, 6]. ACT has become a mainstay of malaria treatment because of its high efficacy and potential to delay the development of anti-malarial resistance [7]. Efficacy and effectiveness studies have shown that ACT treatment enables a recovery rate of over 90% and it is also generally well tolerated [8–10]. ACT is therefore recommended as the first-line anti-malarial treatment strategy in most regions of sub-Saharan Africa [5, 6], although several country-specific studies that focused on ACT supply have found lower availability of ACT and higher availability of monotherapy in both the public and private sector in sub-Saharan Africa [11–13].

In 2010, the WHO instituted a policy recommending parasitological confirmation by microscopy or rapid diagnostic test (RDT) in all cases of suspected malaria prior to treatment, where diagnostic testing is accessible [6], Because the rate between ‘presumptive’ and ‘actual’ parasitological malaria cases can range between 10 and 60% depending on the season, age of patients and transmission area, correct diagnosis of the disease is essential to achieving successful malaria outcomes [14, 15]. Presumptive treatment can accelerate the emergence of parasite resistance and result in non-malarial fevers being treated incorrectly with costly ACT, endangering patient life and quality of care [16].

In most low and middle-income countries access to healthcare for malaria is a key factor in effective disease management, with the private sector representing the majority of service delivery points for malaria treatment [17, 18]. In sub-Saharan Africa, the majority of treatment for malaria is sought outside of the home and outside of the public sector health services. The private providers comprise a wide range of facilities, ranging from health facilities with qualified physicians and nurses, and registered pharmacists, to more general types of retail outlets that often have little or no formal health-related training [19–21]. In many countries, these private retail outlets are often the first, and only, source of malaria treatment [22].

Existing research on the anti-malarial market is typically limited in geographical and country scope. Gaps still remain in understanding the individual factors that influence patient treatment-seeking behaviour and anti-malarial drug purchase decisions. Understanding these factors are essential for developing and implementing better malaria management policies and plans. Furthermore, it is expected that improving healthcare access through both public and private sectors will lead to improvements in the diagnosis and treatment of fevers, regardless of whether or not they are due to malaria or another medical condition.

The objective of this study was to identify patient treatment-seeking behaviour in Ghana, Kenya, Nigeria, Tanzania, and Uganda. Data were collected through nationally representative surveys and included history of illness, diagnostic practices, prescription practices, and drug purchase patterns.

## Methods

### Study sites and sampling strategy

In 2010, in order to address the need for increased access to ACT in both the private and public sector, the Global Fund to Fight AIDS, Tuberculosis and Malaria established the Affordable Medicines Facility-malaria (AMFm). This programme negotiates reduced pricing with ACT manufacturers that provide quality-assured ACT (QAACT) by agreeing to a significant co-payment. AMFm aimed to increase affordability, availability, and use of QAACT in order to crowd out artemisinin monotherapies. AMFm pilot programmes were implemented in eight countries (Cambodia, Ghana, Kenya, Madagascar, Niger, Nigeria, Tanzania and Uganda) [18]. Between April and May 2012, Axios International conducted a study to assess the anti-malarial stock and purchase patterns at private pharmaceutical outlets in five Affordable Medicines Facility-malaria (AMFm) Phase 1 countries: Ghana, Kenya, Nigeria, Tanzania, and Uganda [23]. These five countries were a subset of the eight countries that participate in AMFm and were selected for inclusion because Axios International had partnerships with institutions within them that allowed us to implement and conduct the study in the field. Information regarding private outlets that were recipients of AMFm products is available from several sources, including Novartis International AG, the Global Fund to Fight AIDS, Tuberculosis and Malaria, Population Services International, and in-country distributors [23]. All registered categories of outlets in the private sector were included in the study: private pharmacies, drugs shops, private health facilities and patent medicine vendors (especially in Nigeria). An extensive sampling frame of outlets that are primary recipients of AMFm products in each country was compiled. From this list, approximately 80 outlets per country were selected using random sampling. In order to obtain a set of outlets that were not primary recipients of AMFm products, approximately 20 outlets per country not appearing on the AMFm recipient list were selected by convenience sampling. After examining geographic characteristics of the 100 outlets selected per country, it was determined that the majority were located in urban or semi-urban areas. To better understand the impact of AMFm in rural areas, an additional 20 rural outlets per country were selected. For the sampling of rural outlets, two districts per country were chosen at random from among all districts containing urban outlets that are primary recipients of AMFm products. From each of these districts, convenience and snowball sampling were used to select ten rural outlets, giving a total of 120 outlets per country in Ghana, Nigeria and Tanzania, and 119 outlets per country in Kenya and Uganda [23].

With the objective of assessing patient malaria history and patient treatment-seeking behaviour, a second survey was conducted in the same five countries between September and November 2013. The outlets selected in the first study (April–May 2012) were re-sampled in the second survey. In this second study, an additional area of research on patient treatment-seeking behaviour was added to the research on anti-malarial stock and purchase patterns in the outlets. Between 40 and 45 outlets per country were randomly selected for patient interviews from the sample of 119 and 120 outlets initially selected in the five countries. There was no link between data collected in the outlets and data collected from the patients. Exit interviews were conducted with five patients at each outlet who indicated they had come seeking anti-malarial treatment. A sampling interval of three was used, so that interviews were performed with every third patient who indicated that he/she had come to the outlet seeking anti-malarial treatment. This continued until a total of five patients was reached at each outlet.

### Data collection

Axios International undertook a 2-day training session with data collection field workers, focusing on sampling procedures and questionnaire administration. Field workers were provided with a list of selected clusters and maps that illustrated their administrative boundaries. Participants were eligible for interview if they were seeking anti-malarial treatment. The questionnaire was anonymous, and was structured and standardized for the five countries. It was pre-tested and validated in each country before data collection. The face-to-face interviews were conducted in the appropriate local language. All participants in the study were informed of the voluntary nature of participation and the confidentiality of data collection. All participants were requested to provide verbal informed consent prior to the interviews.

The questionnaire collected data across a range of factors, including: (a) socio-demographic characteristics (age, gender); (b) history of illness (fever and number of days of fever); (c) diagnostic practices (location of malaria diagnosis, type of malaria blood test diagnosis, i.e. microscopy or RDT); (d) prescription practices (did the patient have/not have an anti-malarial drug prescribed by a healthcare worker, type of anti-malarial drug prescription in generic name); and, (e) purchase decision (was the treatment purchased/not purchased) and the reason for the purchase decision. The price paid for the treatment was collected from the outlet vendor where the patient purchased the treatment, and was recorded in the national currency. This price was then later converted into US dollars (US$) and average and median prices were calculated.

### Statistical analysis

Descriptive statistics are given as means with their standard deviation (SD) and median for continuous variables and percentages for categorical variables. Chi square test for qualitative data was used. ANOVA test was used for comparisons of three and more quantitative variables. The concordance between the drugs prescribed and drugs purchased was tested using Kappa test. Variables with p < 0.20 from the univariate analysis were introduced into two logistic regression models in order to evaluate factors associated with availability of malaria drug prescription and to identify factors associated with ACT drug purchases in each country. Adjusted odds ratio (AOR) and 95% confidence interval (CI) were calculated. Associations were considered statistically significant when p < 0.05. Statistical analysis was conducted using Stata 11.0 software package (StatCorp, TX, USA).

### Ethics statement

Participation in this study was voluntary, with all participants providing oral informed consent prior to the interviews. All information was collected anonymously and used solely for research purposes. The Western Institutional Review Board (WIRB) approved the research (#1-904491-1).

## Results

A total of 994 patients were included from the five countries: 199 patients in 45 outlets in Ghana, 195 (41 outlets) in Kenya, 200 patients (40 outlets) in Nigeria, 200 patients (41 outlets) in Tanzania, and 200 patients (41 outlets) in Uganda. In the five countries, the overall ratio of men to women (M:W) was 1.35. The overall mean age was 26.8 years (SD = 15.6) with a significant difference across the five countries: 32.2 years (SD = 13.9) in Ghana, 27.7 (SD = 16.7) in Kenya, 23.7 (SD = 14.1) in Nigeria, 27.1 (SD = 18.4) and 21.0 (SD = 15.4) in Tanzania and 21.0 (SD = 15.5) in Uganda (p < 10^−4^).

Table 1 describes the baseline characteristics of the patients included in the five countries. Location of malaria diagnosis was significantly different in the five countries: 83.4% of patients in Ghana were self-diagnosed whereas 95.5% of patients in Tanzania had a blood diagnostic test and 78.9% in Uganda were diagnosed in a health facility. In Ghana 3.5% of patients had a blood test (100% microscopy), 50.8% in Kenya (67.1% microscopy), 38.0% in Nigeria (65.2% microscopy tests), 95.5% in Tanzania (57.6% microscopy) and 84.5% in Uganda (71.7% microscopy) (p < 10^−4^). In total, 484 blood diagnostic tests were performed, including 315 microscopy tests (72.3%) and 169 RDTs; these blood diagnostic tests were not performed in the outlets at which patients purchased anti-malarial drugs. Of the 484 patients who underwent blood diagnostic testing, 92.3% tested positive.

**Table 1:** Baseline characteristics of patients by country 2013 (N = 994)

	Ghana (n = 199)	Kenya (n = 195)	Nigeria (n = 200)	Tanzania (n = 200)	Uganda (n = 200)	p
Patient characteristics						
Sex ratio M:W	0.69	1.11	0.94	0.96	0.82	0.17
Age (years)						
≤5	17.4	14.2	11.6	12.4	13.1	
6–16	10.6	17.0	13.3	18.9	23.1	0.01
≥17	72.0	68.8	75.1	68.7	63.8	
History of illness						
Presence of fever before attending outlet	98.5	90.0	100	82.0	96.0	p < 10^−4^
Mean days with fever (SD)	2.6 (2.9)	2.5 (1.5)	3.4 (1.8)	2.0 (2.1)	4.1 (5.0)	p < 10^−4^
Location of malaria diagnosis						
Self-diagnosis	83.4	35.5	38.5	2.0	11.3	
Health facility	15.6	49.2	39.5	95.5	78.9	p < 10^−4^
Pharmacy/drug shop	1.0	13.7	20.0	2.5	9.3	
Other	0	1.6	2.0	0	0.5	
Diagnosis practices						
Blood test diagnostic	3.5	50.8	38.0	95.0	84.5	p < 10^−4^
Type of blood test						
Number	6	73	69	184	152	
Rapid diagnostic test	0	32.9	34.8	42.4	28.3	p < 10^−4^
Microscopy	100	67.1	65.2	57.6	71.7	
Positive blood test	100	85.8	94.4	91.1	96.4	0.02
Drug purchased^a^	100	90.0	90.8	92.7	96.5	0.69
ACT purchased^a^	100	96.6	88.4	69.7	55.6	p < 10^−4^
Antimalarial drug prescription and drug purchased						
Drug prescribed	17.6	45.1	34.0	90.0	80.0	p < 10^−4^
Drug purchased	100	86.7	92.0	92.0	96.5	p < 10^−4^
Outlet characteristics						
Type of outlet						
Pharmacy	88.0	100	24.6	87.5	22.5	
Drug shop	6.8	0	7.5	0	18.0	p < 10^−4^
Private health facility	5.2	0	5.0	12.5	59.5	
Patent medicine vendor	0	0	62.9	0	0	

^a^Among patient blood diagnosis tested

A total of 531 patients had an anti-malarial drug prescription (Ghana 17.6%, Kenya 45.1%, Nigeria 34%, Tanzania 90.0%, and Uganda 80.0%) (p < 10^−4^). The concordance between anti-malarial drug prescription and anti-malarial drug purchased was 0.86 in Ghana, 0.87 in Kenya, 0.90 in Nigeria, 0.84 in Tanzania, and 0.91 in Uganda (p < 10^−4^).

Table 2 compares the characteristics of patients with and without an anti-malarial prescription in each country. The overall median price of anti-malarial drugs purchased was US$3.00 in patients with a prescription and US$2.80 in patients without a prescription (p = 0.09). Table 3 summarizes, the predictive independent factors significantly associated with anti-malarial drug prescription after logistic regressions fitted by country.

**Table 2:** Comparison between patients with a drug prescription (Pr+) and patients without a drug prescription (Pr−) by country 2013 (N = 994)

	Ghana			Kenya			Nigeria			Tanzania			Uganda		
	Pr+ (n = 35)	Pr− (n = 164)	p	Pr+ (n = 88)	Pr− (n = 107)	p	Pr+ (n = 68)	Pr− (n = 132)	p	Pr+ (n = 180)	Pr− (n = 20)	p	Pr+ (n = 160)	Pr− (n = 40)	p
Patient characteristics															
Sex-ratio M:W	0.59	0.71	0.22	0.81	1.43	0.05	1.00	0.91	0.09	1.05	0.43	0.07	0.74	1.22	0.16
Age (years)															
≤5	40.0	0.6		19.8	12.5		20.6	13.7		22.9	15.0		26.4	22.5	
6–16	5.7	5.5	<10^−4^	24.4	6.7	0.001	17.6	9.8	0.09	8.4	5.0	0.58	13.2	10.0	0.72
≥17	54.3	93.9		55.8	80.8		61.8	76.5		68.7	80.0		60.4	67.5	
History of illness															
Presence of fever	97.1	98.8	0.56	98.8	82.5	0.001	100	100	–	83.3	70.0	0.14	96.3	95.0	0.71
Mean days with fever (SD)	4.0 (5.4)	2.3 (1.9)	0.002	2.6 (1.6)	2.4 (1.3)	0.56	3.9 (2.1)	3.1 (1.6)	0.002	1.9 (3.9)	3.6 (3.9)	0.002	4.3 (5.4)	3.3 (2.9)	0.24
Diagnostic practices															
Location of malaria diagnosis															
Self-diagnosis	8.6	99.4		1.2	66.0		0	59.7		0	20		1.9	51.3	
Health facility	88.6	0.0	<10^−4^	90.7	15.0	<10^−4^	85.1	17.1	<10^−4^	99.4	60.0	<10^−4^	92.9	21.6	<10^−4^
Pharmacy/drug shop	2.8	0.6		8.1	10.0		14.9	23.2		0.6	20.0		5.1	27.0	
Blood test diagnostic	20.0	0	<10^−4^	94.3	15.0	<10^−4^	79.4	16.7	<10^−4^	99.4	60.0	<10^−4^	96.9	35.0	<10^−4^
Type of outlet															
Pharmacy	82.9	89.1	0.30	100	100	–	34.3	19.7	0.02	86.7	95.0	0.29	20.7	30.0	0.20
Other^a^	17.1	10.9		0	0		65.7	80.3		13.3	5.0		79.3	70.0	
Median price of drug purchased	1.05	1.37	0.008	3.69	1.73	0.04	3.22	2.28	0.04	1.39	1.37	0.87	2.92	3.73	0.30

^a^This category includes drug shops, private health facilities and patent medicine vendors

**Table 3:** Determinants associated with having a prescription in the five countries 2013 (logistic regression) (N = 994)

	AOR	95% CI	p
Ghana			
Price of drug purchased	0.38	0.15–0.94	0.04
Age (years)			
≥17	1.0		
6–16	2.51	0.40–11.50	0.37
≤5	56.8	5.67–89.9	<10^−4^
Mean day of fever	1.10	0.91–1.32	0.31
Kenya			
Price of drug purchased	1.10	0.98–1.24	0.12
Age (years)			
≥17	1.0		
6–16	5.40	1.97–14.83	0.001
≤5	2.63	1.09–6.38	0.03
Presence of fever			
No	1.0		
Yes	5.79	0.40–9.34	0.20
Nigeria			
Price of drug purchased	1.22	1.02–1.45	0.01
Age (years)			
≥17	1.0		
6–16	1.75	0.64–4.77	0.28
≤5	3.59	1.48–8.70	0.005
Mean day of fever	1.33	1.10–1.61	0.02
Tanzania			
Price of drug purchased	1.10	0.66–1.37	0.42
Age (years)			
≥17	1.0		
6–16	1.22	0.14–10.83	0.86
≤5	1.15	0.28–4.76	0.84
Mean day of fever	0.79	0.66–0.94	0.01
Uganda			
Price of drug purchased	0.93	0.79–1.09	0.38
Age (years)			
≥17	1.0		
6–16	1.40	0.43–4.51	0.38
≤5	0.98	0.41–2.35	0.98
Mean day of fever	1.07	0.95–1.20	0.27

Among the 531 patients with an anti-malarial prescription (53.4% of all patients interviewed), 440 (82.9%) had an ACT prescription: 96.9% in Ghana, 97.8% in Kenya, 91.0% in Nigeria, 75.9% in Tanzania, and 61.8% in Uganda had an ACT drug prescription (p < 10^−4^).

A total of 923 patients (92.9%) purchased anti-malarial drugs, and a comparison of the characteristics of patients purchasing ACT or non-ACT drugs is shown in Table 4. Of the 923 patients who purchased anti-malarial drugs, a total of 730 patients (79.1%) purchased an ACT drug (98.0% in Ghana, 90.5% in Kenya, 80.4% in Nigeria, 69.2% in Tanzania, and 57.7% in Uganda) (p < 10^−4^). There were 71 patients (7.1%) who did not purchase an anti-malarial drug, of which 54.4% had a blood diagnosis test and 56.9% did not (p = 0.69). Among the 71 patients (7.1%) who did not purchase an anti-malarial treatment, the primary reason given was: (a) the price was too high (31 patients, 43.7%); (b) the prescribed drug was not available (25 patients, 35.2%); (c) the pharmacist reported that the patient did not have malaria (five patients, 7.0%); and, (d) other reasons (ten patients, 14.1%). ACTs were purchased by 72.5% of patients who had undergone blood testing and 86.5% of patients without a blood test, regardless of whether the test result was positive or negative (p < 10^−4^). Among the 484 patients with a blood diagnosis test, 86.1% of patients testing negative and 92.7% of patients testing positive purchased an anti-malarial drug (p = 0.15).

**Table 4:** Comparisons between patient purchased ACT drugs (ACT+) and patient purchased non-ACT drugs (ACT−) by country (N = 923), 2013

	Ghana			Kenya			Nigeria			Tanzania			Uganda		
	ACT+ (n = 193)	ACT− (n = 4)	p	ACT+ (n = 152)	ACT− (n = 16)	p	ACT+ (n = 148)	ACT− (n = 36)	p	ACT+ (n = 128)	ACT− (n = 57)	p	ACT+ (n = 109)	ACT− (n = 80)	p
Patient characteristics															
Sex-ratio M:W	0.69	0.33	0.52	1.04	1.70	0.38	0.95	0.80	0.65	0.78	1.85	0.008	0.60	1.10	0.04
Age (years)															
≤5	6.2	75.0		16.2	6.3		16.2	11.1		29.1	7.0		21.3	32.5	
6–16	5.2	25.0	<10^−4^	15.5	6.3	0.28	14.2	2.8	0.09	9.4	7.0	0.002	13.9	10.0	0.20
≥17	88.6	0		68.2	87.6		69.6	86.2		61.4	86.0		64.8	57.5	
History of illness															
Presence of fever	98.4	100	0.80	91.0	90.9	0.98	100	100	–	90.6	71.9	0.001	96.3	96.2	0.98
Mean days with fever (SD)	2.4 (2.1)	3.5 (2.5)	0.31	2.5 (1.5)	2.1 (0.7)	0.40	3.4 (1.9)	3.0 (1.6)	0.12	1.9 (1.8)	2.5 (2.8)	0.11	3.2 (3.6)	5.3 (6.5)	0.01
Diagnostic practices															
Location of malaria diagnosis															
Self-diagnosis	84.9	25.0		32.2	61.5		33.3	67.6		0	3.5		12.9	9.3	
Health facility and pharmacy	15.1	75.0	0.001	67.8	38.5	0.03	66.7	32.4	0.0002	100	96.5	0.03	87.1	90.7	0.45
Blood test diagnostic	3.1	0	0.72	55.3	18.8	0.005	41.2	22.2	0.03	96.9	94.7	0.48	81.7	88.7	0.18
Type of outlet															
Pharmacy	88.6	50.0	0.02	100	100	–	27.2	13.9	0.09	87.5	91.2	0.46	29.4	11.3	0.01
Other	11.4	50.0		0	0		72.8	86.1		12.5	8.8		70.6	87.7	
Have a prescription	90.9	0.1	0.001	97.4	2.6	0.006	37.2	19.4	0.04	90.6	92.9	0.60	76.1	86.3	0.08
Median price of purchased drug	1.32	3.69	0.01	2.02	1.67	0.71	3.22	1.24	<10^−4^	2.01	1.14	0.001	4.48	1.14	<10^−4^

The results of multivariate analysis of predictive factors associated with ACT drug purchases are shown in Table 5. The first logistic regression, which included the countries as independent variables, found that country, gender, age, type of outlet, presence of fever, and price of drugs purchased were significant predictors of ACT drug purchasing. Having a drug prescription was not significantly associated with an ACT drug purchase, with the exception of Kenya.

**Table 5:** Predictive factors of ACT drugs purchased according to the country, 2013 (logistic regression) (N = 923)

	AOR	95% CI	p
4 countries			
Country			
Kenya	1.0		
Nigeria	0.43	0.18–1.01	0.05
Tanzania	0.18	0.09–0.39	<10^−4^
Uganda	0.12	0.05–0.28	<10^−4^
Sex			
Women	1.0		
Men	0.66	0.46–0.96	0.03
Age (years)			
≥17	1.0		
6–16	2.39	1.18–4.81	0.01
≤5	1.65	1.02–2.68	0.03
Have a prescription			
No	1.0		
Yes	1.01	0.63–1.61	0.98
Price of drug purchased	1.23	1.12–1.35	<10^−4^
Type of outlet			
Other	1.0		
Pharmacy	1.73	1.01–2.96	0.04
Presence of fever			
No	1.0		
Yes	2.74	1.37–5.50	0.005

	AOR	95% CI	p
Kenya			
Sex	1.67		
Women	1.06	0.32–4.46	0.93
Men			
Age (years)			
≥17	1.0		
6–16	1.48	0.16–13.0	0.21
≤5	2.26	0.26–17.2	0.03
Have a prescription			
No	1.0		
Yes	5.68	1.18–27.3	0.003
Price of purchased drug	0.91	0.81–1.00	0.07
Nigeria			
Sex	1.68	1.25–2.26	0.0006
Women	1.0		
Men	1.57		
Age (years)		0.69–3.54	0.28
≥17	1.0		
6–16	5.10	0.63–23.8	0.13
≤5	2.25	0.67–7.59	0.19
Have a prescription			
No	1.0		
Yes	1.59	0.60–4.20	0.35
Price of drug purchased	1.73	1.28–2.34	0.0004
Type of outlet			
Other	1.0		
Pharmacy	1.50	0.49–4.60	0.47
Tanzania			
Sex			
Women	1.0		
Men	0.40	0.20–0.82	0.01
Age (years)			
≥ 17	1.0		
6–16	2.43	0.62–9.63	0.20
≤5	4.27	1.37–13.34	0.01
Have a prescription			
No	1.0		
Yes	0.73	0.21–2.58	0.63
Price of drug purchased	1.19	1.04–1.35	0.01
Type of outlet			
Other	1.0		
Pharmacy	0.51	0.15–1.79	0.29
Presence of fever			
No	1.0		
Yes	3.14	1.22–8.04	0.02
Uganda			
Sex			
Women	1.0		
Men	0.65	0.31–1.40	0.65
Age (years)			
≥17	1.0		
6–16	1.82	0.56–5.94	0.32
≤5	0.74	0.31–1.76	0.49
Have a prescription			
No	1.0		
Yes	0.59	0.23–1.53	0.38
Price of drug purchased	1.95	1.59–2.40	<10^−4^
Type of outlet			
Other	1.0		
Pharmacy	1.95	0.68–5.58	0.21
Presence of fever			
No	1.0		
Yes	1.40	0.24–8.16	0.71

Table 6 presents a comparison by country of the median prices of ACT drugs purchased with or without a prescription. The price of ACT drugs purchased with a prescription was higher than ACT drugs purchased without a prescription in Kenya (+US$1.96), Nigeria (+US$0.04) and Tanzania (+US$1.41).

**Table 6:** Median price (in US$) of ACT drugs prescribed and purchased, and ACT drugs purchased without a prescription by country 2013

	Ghana (n = 193)	Kenya (n = 152)	Nigeria (n = 148)	Tanzania (n = 128)	Uganda (n = 109)	p
Median price (US$)						
ACT prescribed and purchased	1.05	3.69	3.22	2.69	4.48	
ACT not prescribed and purchased	1.37	1.73	3.18	1.28	–	<10^−4^
p	0.001	0.01	0.22	0.08	–	

## Discussion

This paper presents standardized, nationally representative data on treatment-seeking behaviour for anti-malarial drugs across Ghana, Kenya, Nigeria, Tanzania, and Uganda in 2013. The findings of this study highlight the important role that private sector outlets play in meeting demand for anti-malarial drugs, including WHO-recommended ACT. Of the 994 patients interviewed at private retail outlets in this study, 92.9% purchased anti-malarial medicines, and 79.1% of these patients purchased ACT drugs. It should be noted that purchase of anti-malarial drugs was not dependent on the patient having a confirmed diagnosis of malaria or having an anti-malarial prescription.

These findings are significant for national malaria diagnosis and treatment policy development in low- and middle-income countries, where the private sector delivers approximately 60% of malaria medications to patients [20, 21]. They show that patients can access WHO-recommended ACT treatment when they suspect they have malaria without using public sector healthcare facilities and without having a confirmed diagnosis of malaria and/or an anti-malarial prescription. However, there were significant country-level differences in drug prescription and malaria diagnosis practices across the five sub-Saharan African countries surveyed.

Some 730 patients (79.1% of the patients who purchased anti-malarial drugs) purchased an ACT drug, ranging from 57.7% in Uganda to 98.0% in Ghana. Of the 531 patients who had an anti-malarial drug prescription (53.4% of total patients interviewed), 82.9% had prescriptions for an ACT. While this highlights the high incidence of ACT-recommended treatment for those with an anti-malarial drug prescription, the prevalence of having an anti-malarial prescription (ACT or non-ACT) varied greatly across the five countries, ranging from 17.6% in Ghana to 90% in Tanzania. The authors believe that the high percentage of patients with an anti-malarial prescription in both Tanzania (90%) and Uganda (80%) is due to the high percentage of patients in these countries who received their malaria diagnosis at a health facility (95.5 and 78.9%, respectively).

The type of malaria diagnosis significantly differed across the five countries. In Ghana 83.4% of patients diagnosed themselves with malaria without contact with a healthcare professional, while 95.5% of patients in Tanzania received their diagnosis in a health facility. Not surprisingly, the type and location of malaria diagnosis impacted the number of patients who received a parasitological confirmation by blood test. Although the WHO recommends confirmation of diagnosis with microscopy or RDT in all cases of suspected malaria prior to treatment, the current study found that such tests were performed in only 49% of patients overall, with a large range in the percentage of patients undergoing testing across the five countries included in the study (from 3.5% in Ghana to 95.5% in Tanzania). This finding suggests that the location of diagnosis is an important consideration for national policy makers when designing successful malaria treatment programmes [24].

The study also found that the presence or absence of a prescription was not associated with the price of anti-malarial drugs was not associated with having a prescription Kenya, Tanzania, Uganda. In these countries the price of ACT drugs purchased without a prescription was lower than ACT drugs purchased with a prescription. These results suggest that the price per adult dose in this study is significantly reduced compared with a previous study, which may be attributed to the wide penetration and utilization of AMFm drugs [25]. The prior study found that the purchase price of AMFm ACT drugs was substantially lower than non-AMFm equivalent drugs.

Overall, effective malaria control requires a comprehensive strategy of prevention, correct diagnosis and treatment. The policy of each national health system plays an important role in promoting or hindering access to effective malaria diagnostic services and treatment. Therefore, government should collectively consider the factors mentioned above, including the role of the private sector, access to medication, drug prescription practices, malaria diagnosis practices and drug prices, when shaping health policy around malaria case identification and management.

### Role of the private sector

Some researchers argue that the emphasis of national malaria policies should remain on the public health sector, with the goal of attracting more people to outlets where diagnosis and care are regulated and provided by trained staff [26]. Other studies argue that, given the current state of developing country health systems, the private sector will increasingly work in partnership with more formal health care delivery systems. Therefore, strategies should be adopted to improve quality management within the private sector [27].

There are currently two prevailing strategies for controlling malaria. The first, adopted by the AMFm, favours price subsidies for ACT and RDTs and driving sales through private outlets [23]. A recent study suggested that five years after QAACT implementation, the private sector co-payment mechanism was associated with positive and sustained improvements in QAACT availability, price and market share in Nigeria, Tanzania and Uganda, with more mixed results in Kenya. The authors emphasized the positive results and the important role that the private sector plays in distributing anti-malarial drugs in these countries compared with the private sector [18]. In addition, other studies suggest that the AMFm approach may inform other health interventions aimed at reaching hard-to-reach communities, particularly in the context of universal access to health interventions [28, 29]. Another recent study found that most of the anti-malarial drugs distributed by the private sector were ACT, though one in five anti-malarials distributed was a sulfadoxine–pyrimethamine, which is less expensive than ACT. In many cases, private providers use available malaria commodities to test fever cases and treat according to test results and a recent study showed that the use of RDT in private retail setting increased the referral of patients to other providers, suggesting that these outlets may help drive appropriate care for malaria and other health conditions [17, 30]. Despite these benefits associated with private sector outlets, gaps exist in appropriate case management [17]. However, promoting subsidized medicines in the private sector presents several challenges, including guaranteeing a nationwide affordable price, access for low-income patients and patients in rural areas, and ensuring accurate treatment [16].

The second strategy is the provision of free malaria services through networks of public providers and community health workers. Here, a community health worker network is seen as the most effective channel to deliver malaria treatment in developing countries [16].

The findings of the current study suggest that it is essential for governments to recognize the increasingly important role the private sector plays in improving access to effective malaria case management. This will require public health authorities to develop a working relationship with the private sector, community health workers and civil society to ensure that malaria diagnosis and drug distribution follow recommended best-practice guidelines.

### Use of rapid diagnostic tests (RDTs)

The findings of the current study suggest that households continue to use anti-malarials to treat febrile episodes, with only 49% of patients having a blood test diagnosis. In a survey of nine sub-Saharan African settings, private sector RDT availability was less than 15% for all private for-profit outlets and availability of these tests among pharmacies and drug shops was less than 10% in seven countries [23]. Several potential consequences of low microscopy or RDT use include unnecessary out-of-pocket expenses for households, increased risk of drug resistance through overtreatment with ACT and increased morbidity and mortality when the true source of illness is untreated [31]. Therefore, there is a critical need for expanded use of RDTs to guide appropriate treatment by the private sector in fever case management. In countries where doctors and prescribers are scarce and often overstretched, emphasis should be placed on correct diagnosis through RDT at private retail outlets and pharmacies rather than on ensuring that all patients receive an antimalarial prescription.

A number of other studies have also suggested introducing RDTs into pharmacies given the importance of these outlets in the treatment of malaria in most African settings. Ikwobe et al. found that having an RDT before treatment reduces by 42% the chance of selling anti-malarial drugs with symptoms of uncomplicated malaria in a community pharmacy [32]. An analysis of ten studies from the ACT Consortium found that RDTs were associated with significantly lower ACT prescription (8–69% with RDT interventions compared with 20–100% without RDT interventions), although prescribing was not always in concordance with test results [30]. A cluster randomized trial conducted in Ghana confirmed that providing RDTs in the private retail sector significantly reduced the dispensing of anti-malarials to patients without malaria, did not reduce prescribing of anti-malarial drugs to true malaria cases, and appeared safe. This study concluded that RDTs should be considered for the informal private drug retail sector [33]. Other studies estimated that the use of RDTs resulted in a 77–96% reduction in anti-malarial prescriptions [32, 34, 35]. Several studies have found that implementing an RDT in registered drug shops improved appropriate treatment of malaria with ACT compared with presumptive treatment of fevers [36–38]. A recent systematic review shows that private outlets in different countries have the potential to target anti-malarial drugs more effectively, and that they can incorporate RDTs into their practice, although with varying degrees of uptake and influence on case management. Although intensive interventions generally produced better outcomes, it remains unclear whether such efforts could be maintained or scaled up to a national level [39]. Zikusooka et al. suggested that restricting anti-malarial treatment to only RDT-positive patients would save up to US$2.12 per person, if RDT and ACT were sold at governmental price [40].

However, discussions about the potential role of the private sector in the diagnosis and treatment of malaria are hampered by a lack of evidence and the presence of strong and varying opinions among different stakeholders. Despite some positive trial results, major challenges remain in ensuring effective supervision and regulation for both accredited and non-accredited retailers. It is clear that increasing access to RDTs in the private pharmacy will be more challenging than scale-up of ACT availability. National regulatory frameworks that prohibit retail providers from performing consultations and offering an RDT service pose an additional barrier to making RDTs more widely available. This is a barrier that should be overcome, especially in light of a recent study showing that retail providers are willing to incorporate RDTs into their business and that customers are more likely to accept testing before requesting treatment in private pharmacies [41, 42].

### Study limitations and further research

There are some limitations to this study. First, information related to patient characteristics, history and diagnosis of malaria was based on responses of patients and outlet data collection. However, we believe that the use of a standardized methodology in the five countries minimized potential recall bias. Second, the care-seeking behaviour analysed from this sample may not reflect behaviour of more marginalized populations that do not patronize shops that carry anti-malarial drugs, or extremely sick patients who are unable to visit a retail outlet. Third, adult equivalent treatment dose estimations were used when calculating the price of treatments. This calculation has the advantage of allowing direct comparison between drugs and countries. However, it should be noted that in practice some patients obtain drugs for children or purchase incomplete doses. In this study, children and adults were separately identified, which is expected to minimize bias. Fourth, an overestimated, non-representative sample of outlets at least in one country, particularly in urban areas, cannot be excluded as another potential limitation. Additionally, the availability of drugs and range of malaria diagnostic test could be less robust in rural areas. There was an attempt to mitigate these potential limits with the inclusion of 20 additional rural outlets in the final sample. Finally, because drug price data were collected in 2013 it is likely that current pricing information has changed significantly over the past 4 years [18].

This study highlights key areas for further research including provider prescribing practices, and factors influencing providers’ behaviour and perceptions. More qualitative studies are also needed to explore and understand barriers to accessing diagnostic and treatment services. Without a clear understanding of the socio-cultural rationales that drive treatment-seeking patterns, irrational drug use and provider behaviour, effective case management and achieving prompt access will remain a challenge. Such studies should be designed to explore all dimensions of access and how they interrelate at the demand, supply and policy levels [31]. Further research is also needed to better understand how RDT results are being perceived and used by providers and patients, particularly in the private sector, and what steps are being taken to ensure appropriate storage and quality assurance of RDTs [42].

## Conclusions

This multi-country study highlights the different drug prescription and purchase patterns in five sub-Saharan African countries. Two patterns of patient treatment-seeking behaviour for fever and suspected malaria emerged: high attendance at a public healthcare facility prior to visiting a retail outlet in Tanzania and Uganda, and low attendance at a public healthcare facility prior to visiting a retail outlet in Ghana, Nigeria and Kenya. This correlated with higher rates of anti-malarial prescriptions in Tanzania and Uganda compared with Ghana, Nigeria, and Kenya.

Considering this treatment-seeking and diagnostic behaviour, there is a need for improved targeting of the general population and the private sector retail outlets in terms of utilizing RDT diagnosis for effective malaria treatment, and improving education on the effective use of anti-malarial drugs. Due to the increasingly important role the private sector plays in fever case management in sub-Saharan Africa, developing a better understanding of private retail outlet characteristics is critical to designing effective malaria interventions that support public health objectives.
